# Genome-wide DNA methylation profiles reveal novel candidate genes associated with meat quality at different age stages in hens

**DOI:** 10.1038/srep45564

**Published:** 2017-04-05

**Authors:** Meng Zhang, Feng-Bin Yan, Fang Li, Ke-Ren Jiang, Dong-Hua Li, Rui-Li Han, Zhuan-Jan Li, Rui-Rui Jiang, Xiao-Jun Liu, Xiang-Tao Kang, Gui-Rong Sun

**Affiliations:** 1 College of Animal Science and Veterinary Medicine, Henan Agricultural University, Zhengzhou, 450002, China; 2 Henan Innovative Engineering Research Center of Poultry Germplasm Resource, Zhengzhou 450002, China

## Abstract

Poultry meat quality is associated with breed, age, tissue and other factors. Many previous studies have focused on distinct breeds; however, little is known regarding the epigenetic regulatory mechanisms in different age stages, such as DNA methylation. Here, we compared the global DNA methylation profiles between juvenile (20 weeks old) and later laying-period (55 weeks old) hens and identified candidate genes related to the development and meat quality of breast muscle using whole-genome bisulfite sequencing. The results showed that the later laying-period hens, which had a higher intramuscular fat (IMF) deposition capacity and water holding capacity (WHC) and less tenderness, exhibited higher global DNA methylation levels than the juvenile hens. A total of 2,714 differentially methylated regions were identified in the present study, which corresponded to 378 differentially methylated genes, mainly affecting muscle development, lipid metabolism, and the ageing process. Hypermethylation of the promoters of the genes *ABCA1, COL6A1* and *GSTT1L* and the resulting transcriptional down-regulation in the later laying-period hens may be the reason for the significant difference in the meat quality between the juvenile and later laying-period hens. These findings contribute to a better understanding of epigenetic regulation in the skeletal muscle development and meat quality of chicken.

As the second most consumed meat all over the world, poultry plays an important role in people’s lives, and chicken is also one of the main protein sources^1^. For the past few years, the meat quality of chicken has attracted more and more attention^2^,^3^,^4^. Meat quality is affected by many factors, including genetics, nutrition, and feeding environment^5^,^6^,^7^,^8^. Intramuscular fat (IMF) is an important factor influencing meat quality^9^,^10^. Fat is the precursor of flavour substances in meat, and the IMF content contributes to the juiciness and tenderness of the meat^11^. The water holding capacity (WHC), which is always measured with the dropping losses, is closely associated with the juiciness of the meat, and the loss of water is always accompanied by a loss of nutrients in the meat^12^. In addition, the shear force (SF) is a measurement parameter for the tenderness of meat. A weak SF is a good indicator of meat tenderness^13^. Until now, extensive whole-genome association studies have identified many genes affecting the IMF content, WHC and SF of chicken^12^,^14^,^15^. However, the epigenetic molecular mechanism underlying meat quality remains poorly understood.

In recent years, epigenetics has attracted great attention. Epigenetic mechanisms, including DNA methylation^16^, histone modification^17^, non-coding RNAs^18^, and chromatin remodelling^19^, have been demonstrated to be involved in the development of a variety of biological processes. DNA methylation may affect gene expression without changing the DNA sequence and has been found to be closely related with the formation of some complex phenotypic traits^20^,^21^,^22^ and the occurrence and development of various diseases^23^. Previous studies have suggested that DNA methylation contributes to chicken domestication^24^, spermatogenesis^25^, growth development^26^, the ageing process^27^, and disease resistance^20^. Recently, multiple studies have been conducted to identify the genome-wide methylation profiles of economically important animals^21^,^22^,^28^,^29^. With the development of techniques for sequencing entire genomes, a single-base high-resolution methylation sequencing approach, whole genome bisulfite sequencing^30^ (WGBS), has been widely used in studies of DNA methylation associated with specific phenotypes^31^, growth and development^32^, disease^33^, etc.

Gushi hens, as a well-known local breed in southeast China, have a slower growth rate than commercial broilers (e.g., AA broiler and Recessive White Rock). Gushi hens possess a fleshy delicate, special flavour that is abundant in nourishment and have a higher IMF content compared to commercial broilers, and they are becoming increasingly popular among Chinese people^34^,^35^. However, there have long been controversies about the meat quality and the nutritional value between juvenile and old-aged hens^36^,^37^. Over the past few years, most studies have focused on the skeletal muscle of chicken before sexual maturation^38^,^39^,^40^. However, until now, the epigenetic mechanisms responsible for breast meat quality between the juvenile and later laying-period hens remained poorly understood. In preliminary studies, we found a remarkable difference in the meat quality between the two age stages of Gushi hens. The juvenile (20 weeks old) and later laying-period (55 weeks old) hens differed in their skeletal muscle characteristics and their physiological and biochemical indexes. Furthermore, we measured the transcriptome of the breast muscle in the different age groups (Jiang *et al*. 2016, unpublished data) and identified some differentially expressed genes (DEGs) that were closely related with energy and protein metabolism, lipid metabolism, skeletal muscle cell differentiation and meat quality parameters between the juvenile and later laying-period hens. Therefore, we speculated that DNA methylation might contribute to the difference in meat quality between the two age stages.

The aim of the present study was to investigate the potential regulatory roles of DNA methylation affecting the differences in breast meat quality between the two age stages through regulating the expression of related genes. Whole genome single-base DNA methylation profiles of the two different age groups were generated using WGBS. In parallel, combined with the transcriptome sequencing data, the present study aimed to describe the genome-wide DNA methylation patterns in chicken muscle and integrate the relationship between DNA methylation and transcriptional regulation on a genome-wide scale to reveal the novel methylated candidate genes associated with breast meat quality in chicken at the different age stages. The results of this study may contribute to our understanding of the improvement of poultry meat quality and provide some basis and reference data for the study of genomic epigenetics in poultry.

## Results

### Differences in meat traits and serum lipid levels between juvenile and later laying-period Gushi hens

**Figure 1:**
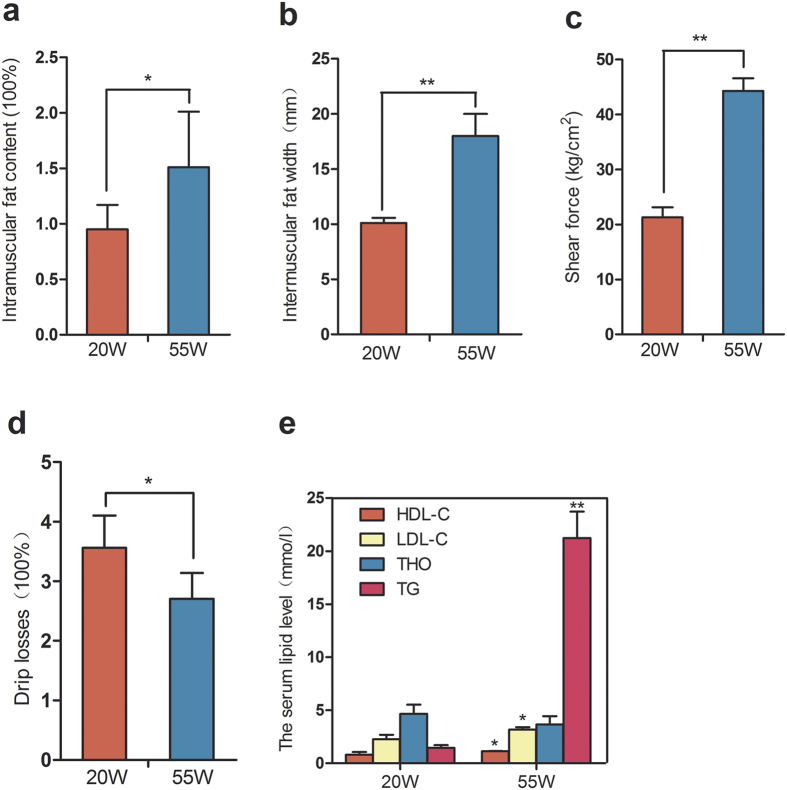
Differences in meat traits and serum lipid level between juvenile (20 W) and later laying-period (55 W) Gushi hens. (**a**–**d**) Intramuscular fat (IMF) content, intermuscular fat wide, shear force (SF), drip losses in breast muscle of 20 W and 55 W; Student’s paired t-test (n = 6). Values are means ± SD. (**e**) The serum lipid level of 20 W and 55 W. Student’s paired t-test (n = 6). Values are means ± SD. *For P < 0.05, and **for P < 0.01.

To confirm the differences in the meat traits and lipid metabolism levels between the juvenile and later laying-period Gushi hens, we measured the IMF content, the intermuscular fat width (IFW), the SF, the dripping losses and the serum lipid levels. The later laying-period hens exhibited a higher IMF content and IFW than the juvenile hens (*P* < 0.05) (Fig. 1a,b). Consistent with our expectations, the later laying-period hens displayed a higher serum lipid level than the juvenile hens. For instance, the triglyceride (TG), high-density lipoprotein cholesterol (HDL-C) and low density lipoprotein cholesterol (LDL-C) levels in the serum were significantly increased (*P* < 0.05), especially the TG level, which showed a 14.74-fold change, whereas no significant difference in the total cholesterol content (THO) (*P* = 0.064) (Fig. 1e). These results suggested that fat deposition ability increased and the lipid metabolism ability decreased with age in Gushi hens. The later laying-period hens had a greater muscle fibre diameter and a smaller density than the juvenile hens (Supplementary Table S1). In addition, compared to the later laying-period, the juvenile hens showed a lower SF but higher drip losses (*P* < 0.01 and *P* = 0.01, respectively) (Fig. 1c,d). These results suggested that the juvenile hens possessed a lower muscle WHC but a higher muscle tenderness than the later laying-period hens. Furthermore, the fatty acid and amino acid compositions in the two age stages were also measured in this study. Although the result did not suggest distinct differences on the whole, the later laying-period hens showed a higher content of unsaturated fatty acids (UFA) than the juvenile hens, especially for C18:1 (42.27% and 37.63%, respectively) (*P* < 0.01) (Supplementary Table S2). In addition, compared with the later laying-period hens, the juvenile hens exhibited a higher content of leucine in the breast muscle tissue (Supplementary Table S3).

### Global mapping and statistical analysis of the WGBS reads

**Figure 2:**
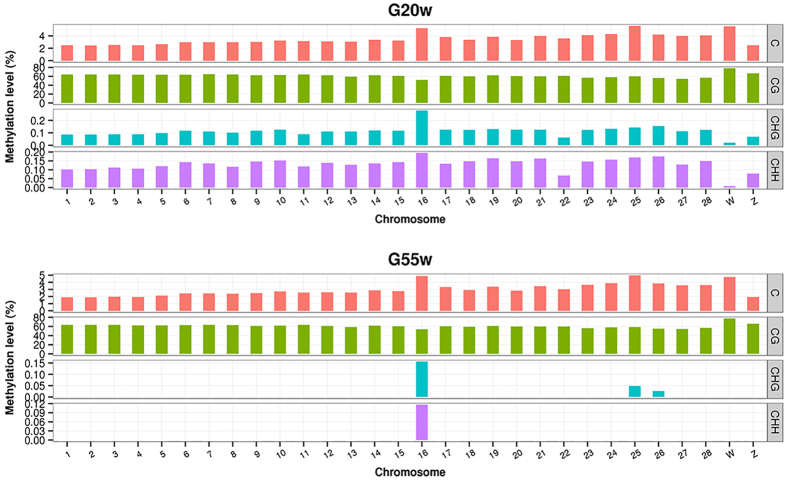
Chromosome distribution of methylation level in various sequence contexts in two groups. The distribution of methylation level in chromosomes 1–28 and the Z, W chromosome of the chicken genome were shown in histogram for each sample. mC signifies 5-methylcytosine. H = A, C, or T. Chromosome numbers and scales are indicated on the periphery (mCG level = mCG/CG).

In the present study, three breast muscle tissues were used to generate one pooled DNA sample for each group of 20-week- (20 W) and 55-week-old (55 W) Gushi hens. A total of 34.43 G and 35.29 G raw data were generated for the two groups, respectively. After removing the low quality reads, 271,942,082 and 279,988,624 clean reads were generated for the two groups, respectively. In the 20 W and 55 W groups, the uniquely mapped reads covered 75.09% and 78.28% of the chicken genome, respectively (Table 1). The DNA methylation level in the various sequence contexts (mCG, mCHG and mCHH) of all of the chromosome regions were statistically analysed and displayed as histograms (1–28 and the Z, W chromosome; Fig. 2). The present study suggested that a methylation loss on a whole-genome scale in a non-CpG context occurred during the ageing process.

### Global DNA methylation patterns of chicken

**Figure 3:**
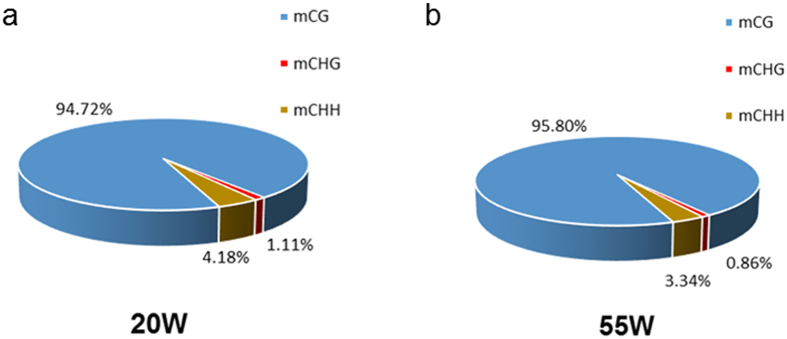
Comparison of DNA methylation patterns in the two groups.

To study the global DNA methylation profile differences between the two groups, we analysed the DNA methylation level on a genome-wide level. Approximately 3.25% and 3.26% of all genomic C sites were methylated in the two groups, respectively. In addition, the research demonstrated that methylations existed in three contexts: CG, CHG, and CHH (where H is A, C, or T). We observed 63.28% CG, 0.3% CHG, and 0.32% CHH methylations in the 20 W group and 63.09% CG, 0.55% CHG, and 0.56% CHH methylations in the 55 W group (Table 2). Among the methylated cytosines, there were 94.72% methylated CG in the 20 W group and 95.80% methylated CG in the 55 W group, which increased by 1.08% from 20 to 55 weeks. However, the rates of methylated CHG and methylated CHH decreased from 1.11% to 0.86% and 4.18% to 3.34%, respectively (Fig. 3). Furthermore, in order to identify the relationship between the sequence context and the methylation preference in the present study, we analysed the methylation level of 9 bases around the methylated cytosine. Interestingly, this study demonstrated an obvious difference in non-CG contexts, especially in the CHH context (Supplementary Fig. S1).

### DNA methylation levels of different functional regions

**Figure 4:**
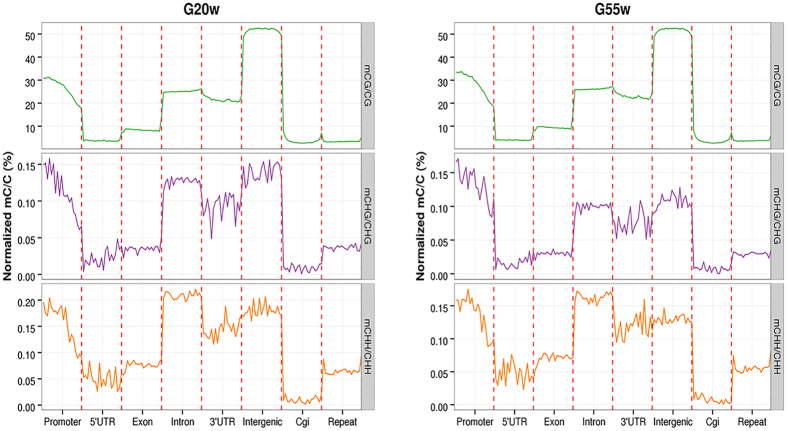
DNA methylation levels of different functional regions between the breast muscle of the two groups.

To decipher the genome-wide DNA methylation profile differences between the 20 W and 55 W groups, we analysed the distribution of DNA methylations in the different genomic regions (Fig. 4). Overall, differential methylation levels in the different functional regions of the genome were observed, especially in the region of the gene promoter (the promoter region was defined as an area 2-kb upstream of the transcription start sites). Among all of the classes, the methylation level of the CpG Island (CGI) was the lowest, followed by the region of repeat and the 5′UTR. The methylation level of the intergenic region was the highest in the two groups.

### Differentially methylated regions (DMRs)

**Figure 5:**
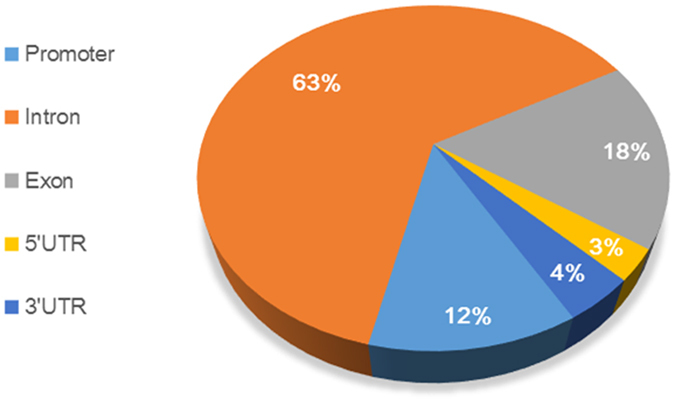
The distribution of DMR regions. DMR, differentially methylated region.

**Figure 6:**
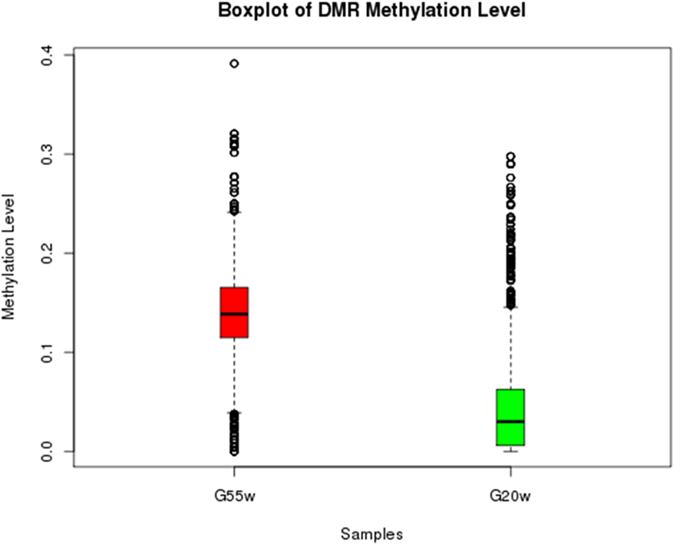
Methylation levels of DMRs in two groups. Boxes, quartiles 25–75%; black lines within boxes, median of the distribution (quartile 50%). DMR, differentially methylated region.

To recognize the regions with different methylation levels in the genomes between the two different age stages, the sliding window method was used to scan each site’s methylation information in the present study. A total of 2714 DMRs were detected (corrected *p*-value < 0.05). Furthermore, we also observed that the methylation levels were negatively correlated with the chromosome length (Pearson’s r = −0.652, *p* < 0.001) and the gene density (Pearson’s r = −0.675, *p* < 0.001) and were positively correlated with the GC percentage (Pearson’s r = 0.948, *p *< 0.001) and that the length distribution of the DMRs tended to be a normal distribution (Supplementary Fig. S2). The DMRs were mainly located in the introns, whose proportion exceeded 60%, followed by the exons and the promoter region (Fig. 5). The boxplot of the DMR methylation levels suggested that the average methylation level of the 55 W group was higher than the 20 W group (Fig. 6). The DMRs that overlapped with the specific gene functional elements were defined as differentially methylated genes (DMGs). A total of 358 DMGs were identified. Of these DMGs, 334 genes were up-methylated, and 45 genes were down-methylated (Supplementary Table S4).

### Functional enrichment analysis of the DMGs

**Figure 7:**
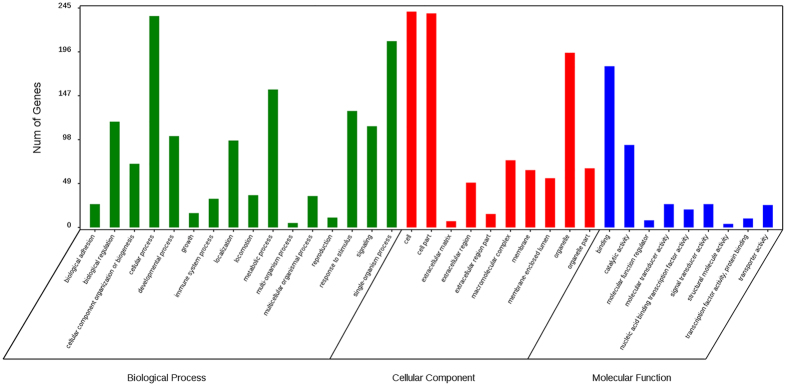
GO (Gene Ontology) categories enriched for genes with DMRs.

**Figure 8:**
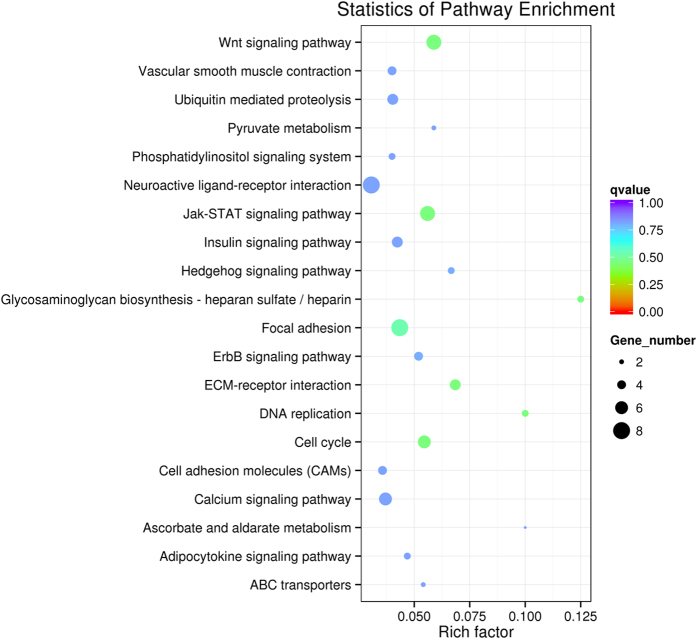
Scatter plot of the top 20 KEGG enrichments. The abscissa represent the richness factor, the ordinate represent the enriched pathway terms. Q-value represents the corrected P, and a small Q-value indicates high significance.

**Figure 9:**
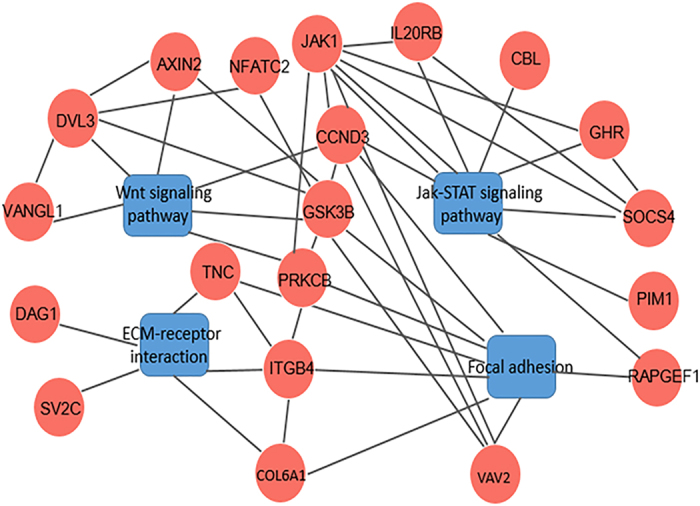
KEGG enrichment pathways and protein-protein interaction network analysis of DMGs.

To investigate the potential biological functions of the DMGs, a gene ontogeny (GO) enrichment analysis and a KEGG pathway analysis were performed. All of the DMGs were annotated in three categories as follows: biological process; cellular component; and molecular function. We noticed that some of these DMGs were enriched in the following biological process terms: cellular process (236; 70.66%); single-organism process (208; 62.28%); and metabolic process (148; 44.31%). In addition, some of the DMGs were enriched in the following cellular component terms: cell (241; 72.16%); cell part (239; 71.56%); and organelle (229; 68.56%). Furthermore, some of the DMGs were enriched in the following molecular function terms: binding (180; 53.89%) and catalytic activity (42; 12.57%) (Fig. 7). We noticed that several genes were involved in the biological processes significant for skeletal muscle development and lipid metabolism, such as *MYO18B, MYO1D, MYO5A, MYOG, ACSS1, ABCA1, ABCC4, ATP11C, ATP13A2, ATP13A5*, and *LRP8* (Supplementary Table. S4). A pathway enrichment analysis identified 87 pathways, and the top 20 pathways are shown in Fig. 8. Of these pathways, some were associated with muscle development and meat quality, such as the Wnt signalling pathway (Q-value = 0.45), the Jak-STAT signalling pathway (Q-value = 0.45), ECM-receptor interaction (Q-value = 0.45) and focal adhesion (Q-value = 0.53). There were 25 differentially methylated genes in these four pathways. Furthermore, the protein-protein interaction network analysis showed that these DMGs were highly correlated with each other (Fig. 9). Notably, the *COL6A1, GHR, SOCS4*, and *JAK1* genes were observed in these pathways. These DMGs, which were involved in muscle development and IMF deposition, might significantly contribute to the meat quality differences at the different age stages.

### Association analysis between the DMGs and the differentially expressed genes (DEGs)

**Figure 10:**
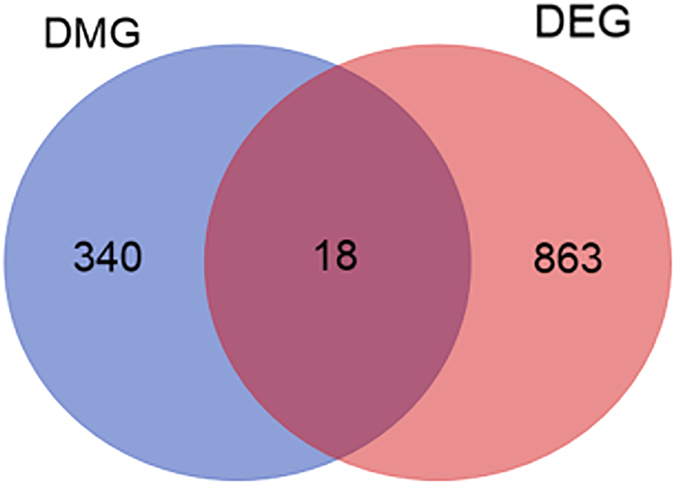
The DMGs that overlapped with DEGs in the two groups. DMG, differentially methylated gene; DEG, differentially expressed gene.

To explore the relationship between DNA methylation and gene expression, we integrated the DMGs with the differentially expressed genes (DEGs) obtained from the chicken breast muscle transcriptome data (Jiang *et al*. 2016, unpublished data). According to the level of gene expression (FPKM, the fragment per kilobase of exon model per million mapped reads), we classified the genes of each sample into two classes, low-expression genes and high-expression genes. We calculated the methylation level of each gene in five functional elements (Supplementary Fig. S3). Compared with the low-expression genes, the high-expression genes showed apparent differences in the methylation level between the two groups, especially in the promoter regions of both CG and non-CG contexts. Furthermore, visible differences in the methylation levels were observed between the two groups in the 5′UTR regions, the Exon regions, the intron regions and the 3′UTR regions of the non-CG contexts. Furthermore, eighteen DMGs overlapped with the DEGs that were identified in the present study (Fig. 10 and Table 3).

### Candidate DMGs associated with meat quality between the two different age stages

**Figure 11:**
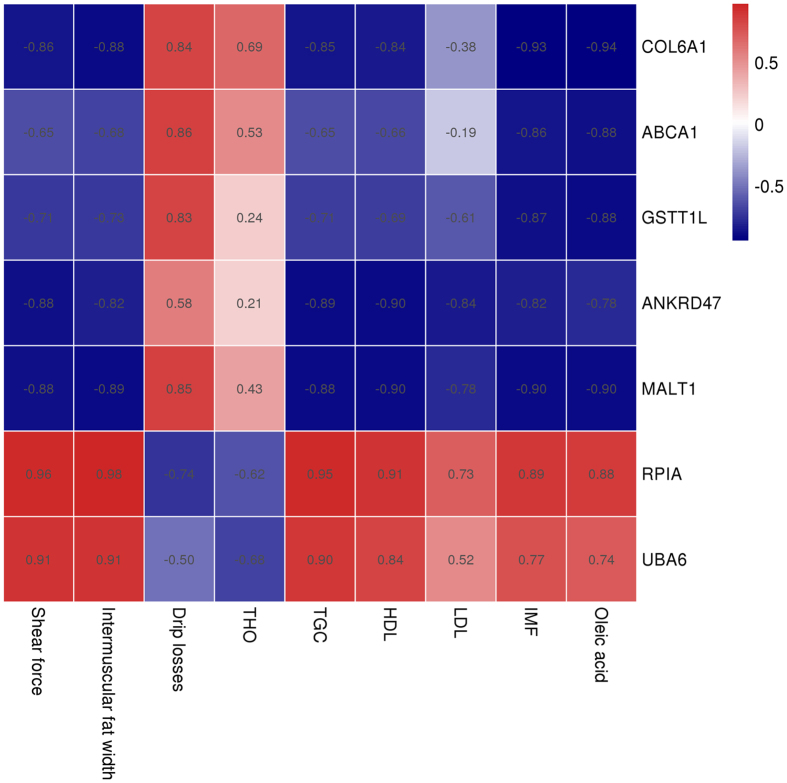
Pearson’s correlations between gene transcriptional level and phenotypic traits of breast muscle at two age stages. The transcriptional level was calculated by FPKM (the fragment per kilobase of exon model per million mapped reads) of each gene. Phenotypic traits parameters: intramuscular fat content, intermuscular fat width, shear force, dripping losses and serum lipid level, oleic acid content.

**Figure 12:**
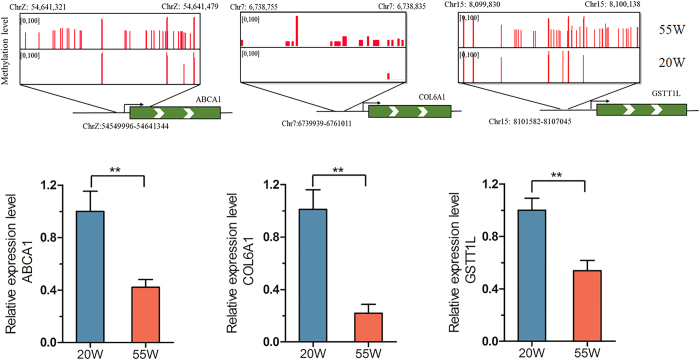
Differentially methylated promoters of *ABCA1, COL6A1, GSTT1L* involved in the difference of meat quality between different age stages. For upper panels, the red column depict methylation level of all mCpG. The black rectangle mark the boundaries of the identified DMR. Lower panels display relative expression levels of genes in 20 W and 55 W. Gene expression levels were detected by Q-PCR and normalized to the expression levels in high one. Student’s paired t-test (n = 3). *For P < 0.05, and **for P < 0.01.

In view of the many genes related to meat quality that have been well explored and studied, the present study analysed the correlations between the transcriptional level and phenotypic traits of breast muscle in the two age stages (Fig. 11). Among the 18 differentially expressed DMGs, the promoters of seven genes were differentially methylated. We noticed that the expression levels of *COL6A1, ABCA1, GSTT1L, ANKRD47*, and *MALT1* were negatively correlated with the IMF, SF, IFW, TGC, and HDL-C and were positively correlated with drip losses. Furthermore, among the seven genes, we found that the promoters of the genes *ABCA1, COL6A1*, and *GSTT1L* were hypermethylated in the 55 W group. In addition, the transcriptional levels of three differentially methylated genes were all down-regulated (Fig. 12).

### Validation of the WGBS data by bisulfite sequencing PCR (BSP)

To validate the reliability of the WGBS data, we used the bisulfite sequencing (BSP) approach. In this study, two hyper DMR and one hypo DMR were randomly selected in the chicken genome. The bisulfite sequencing results were basically consistent with the WGBS data (Supplementary Figure S4), and this indicated that the methylation data obtained by the WGBS was reliable.

## Discussion

Chicken is one of the most important protein and energy sources for the human diet. Chicken meat quality is significantly affected by fat metabolism^9^. Different from mammals, avian adipose tissue has little ability to synthesize fatty acids, and the main organ for poultry fatty acid synthesis is the liver tissue^41^. In poultry, the body fat is mainly deposited in the subcutaneous tissue, visceral tissues (abdominal, intestines and stomach), muscle, bone and other tissues. In chicken, IMF is located throughout the skeletal muscle and not as discrete adipose deposits^9^. The development of fat tissue as well as fat deposition in poultry, depends on the triglyceride levels in the plasma. Many factors influence the deposition of IMF, and a change in the IMF content is associated with different breeds and age stages^42^,^43^. Compared with juvenile hens, the IMF, TG and UFA content of the later laying-period hens was higher, which was consistent with previous research^9^. Nevertheless, the results indicated that the diameter of the muscle fibre increased as the age of the chicken increased, which would result in the meat tenderness getting worse. This may partly explain the reason why the old-aged hens have more flavour but are tougher than juvenile hens.

It is believed that DNA methylation, especially in the promoter regions, usually affects gene expression via different modes^44^. Our results indicated that DNA methylation level decreased dramatically before the TSS and increased towards the gene body regions in chicken (Fig. 4), which was consistent with the results by Li *et al*.^29^. Furthermore, we found that the intron regions of the chicken genome comprised a large proportion of the DMRs (>60%), and only a small proportion of the DMRs were located in the 5′UTR, 3′UTR, and promoter regions (Fig. 5). Further studies focusing on the methylation of the intron regions are needed to elucidate the complicated epigenetic mechanism underlying development in chickens.

Li *et al*.^20^ first established single-base resolution DNA methylation profiles in chicken tissues, and their results suggested that a total of 96.24% of all methylcytosines occurred in the CG context, 0.86% in the CHG context, and 2.89% in the CHH context. In the present study, although a very small proportion (4–5%) of mCH was found in the chicken genome, the methylation level distinctly decreased on a whole-genome scale in the later laying-period hens compared to the juvenile hens (Figs 2 and 3). In addition, the base following a non-CG methylcytosine was almost always an adenine (Supplementary Figure S1), while thymine was observed less often, which was consistent with the result by Li *et al*.^20^. A previous study showed that asymmetric non-CpG methylation always occurs in introns and some repeat elements and that non-CpG methylation might affect DNA thermal stability^45^. Our data showed that a distinct difference in non-CpG methylation existed between the two age groups, and, to some extent, this was related to the global methylation loss during the ageing process. This methylation might affect the transcription of genes related to muscle development and meat quality. Further research is needed to explore the function of non-CpG methylation, which might contribute to understanding the biological significance of the asymmetric non-CpG methylation changes during the ageing process.

The growth and development of skeletal muscle occurs along with changes in the muscle fibre diameter and number^38^. In the present study, we identified some DMGs associated with muscle growth and development, including myosin XVIIIB (*MYO18B*), myosin ID (*MYOD*), myosin VA (*MYO5A*), myogenin (myogenic factor 4) (*MYOG*), and fibroblast growth factor 12 (*FGF12*). For example, *MyoD* is one of the four members of the myogenic regulatory family, and with the aid of other factors, *MYOD* acts on the promoter region or enhancer region of many genes to promote their transcriptional activity and is involved in the proliferation and differentiation of muscle satellite cells^46^. Zhang *et al*.^47^ reported that single nucleotide polymorphisms (SNPs) in the MyoG and Myf5 genes were associated with chicken growth traits. Furthermore, we identified several DMGs involved in encoding collagen, type XIII, alpha 1 (*COL13A1*), collagen, type XX, alpha 1 (*COL20A1*), collagen, type VI, alpha 1 (*COL6A1*), ADAM metallopeptidase with thrombospondin type 1 motif, 2 (*ADAMTS2*) and some calmodulin-related genes. The main component of connective tissue is collagen, and the content and properties of collagen are closely related with the tenderness of muscle. The non-reducing bridging structure between the collagen increases with age, which then affects the quality and texture of the meat^48^. It was suggested that the *COL6A1* gene is important for cell adhesion and is also related to the extracellular matrix (ECM)^49^. The ECM is a part of three connective tissue layers surrounding muscle fibres^50^. The ECM is composed of fibrous and non-fibrous proteins, including collagens and proteoglycans. Overall, there might exist a relationship between the expression of genes related to collagen encoding and the ECM and meat quality traits. *ADAMTS2* as a class of Zn^2+^-dependent secretory metalloproteinases and is a key shear enzyme in the formation of collagen that plays an important role in the process of forming collagen and indirectly regulates the deposition of IMF, SF and other meat quality traits^51^. Moreover, Yang *et al*.^52^ found that calcineurin *(CaN*) and Ca^2+^/calmodulin-dependent protein kinase (*CaMK)* had different effects on adipogenesis in the muscle of chickens.

Furthermore, as IMF is located throughout the skeletal muscle and is not a discrete adipose deposit in chicken, it is necessary to systematically study the DEGs related to muscle development or lipid metabolism in current study, which would contribute to understanding the deposition of IMF in the muscle tissue. Our previous transcriptome analysis indicated that several DEGs, between the two age groups, are involved in glycerophospholipid and glycerolipid metabolism, steroid biosynthesis, fatty acid elongation and degradation, such as *HADHA, HADHB, ACAA2, ALDH3A2, LPCAT2, PLA2G12A, PLIN1, CYP27A1, AGPAT3, AGPAT9, CETP*, and *PPARGC1B,* and some are differentially expressed long non-coding RNAs (lncRNAs) (unpublished data). In the present study, we also identified some differentially methylated genes, including ATP-binding cassette, sub-family A, member 1(*ABCA1*), acetyl-CoA short chain synthetase 1 (*ACSS1*), low density lipoprotein receptor-related protein 8, apolipoprotein e receptor (*LRP8*), solute carrier family 33 (acetyl-CoA transporter), member 1 (*SLC33A1*), solute carrier family 44 (choline transporter), and member 1 (*SLC44A*1), which are associated with lipid metabolism. Adipose tissue contains one of the largest reservoirs of cholesterol in the body^53^, and *ABCA1* plays a major role in cholesterol efflux, maintaining cholesterol homeostasis and lipid metabolism in adipocytes^53^. In this study, the promoter of the *ABCA1* gene was hypermethylated in the later laying-period hens, and the hypermethylated promoter of *ABCA1* in the later laying-period hens caused a significant decrease in *ABCA1* expression. *ACSS1* plays a key role in glycolysis for energy production and is also a key enzyme in the activation of short chain fatty acids through the formation of thioesters with CoA^54^. A previous study demonstrated that *LRP8* participates in the removal of cholesterol and, thus, plays a crucial role in maintaining lipid homeostasis^55^.

Previous studies indicated that increased protein catabolism occurs in ageing skeletal muscles^27^,^56^. The ubiquitin-proteasome system (UPS) is the main pathway of protein degradation in cells^57^. Our results suggested that some differentially methylated genes were associated with ubiquitin modification, including ubiquitin-like modifier activating enzyme 6 (*UBA6)*, ubiquitination factor E4A *(UBE4A)*, ubiquitin protein ligase E3 component n–recognin 4(*UBR4)* and ubiquitin specific peptidase 5 (*USP5*). The results indicated that DNA methylation might affect the expression of these ubiquitin-related genes and, thus, participate in protein degradation in breast muscle. In addition, a previous study demonstrated that some *USP* genes were strongly correlated with the WHC, and therefore, these genes might be important in maintaining moisture in the meat^12^. The *TNC* gene is a member of a family of genes coding for extracellular matrix proteins^58^, and it plays an important role in cell communication, extracellular matrix receptor interactions and focal adhesion.

Interestingly, we found several DMGs related to glutathione metabolism, which is suggested to be associated with antioxidant and senescence, including glutathione S-transferase theta 1-like (*GSTT1L*), gamma-glutamyl transferase 1 (*GGT1*), glutamate receptor, ionotropic, N-methyl-D-aspartate 3 (*AGRIN3A*), and guanine monophosphate synthase (*GMPS*). A previous study found these genes are related with the size of the loin eye area (LEA) in cattle^12^. Furthermore, we found that the promoter of *GSTT1L* was hypermethylated in the later laying-period hens and that the mRNA expression level was lower than in the juvenile hens. Our result suggested that *GSTT1L* might contribute to the differences of poultry meat quality in the different age stages.

In the present study, energy metabolism-related genes were differentially methylated, e.g., glycogen synthase kinase 3 beta (*GSK3B)*, protein kinase, AMP-activated, gamma 2 non-catalytic subunit (*PRKAG2)*, protein kinase C, beta (*PRKCB*) and calpain 5 (*CAPN5*). The results of a recent study by Jin *et al*. showed that *PRKAG2* is associated with feed intake (FI) and body weight (BW) in chicken^59^. *CAPN5* encodes a proteolytic enzyme involved in the rate of proteolytic changes in cells, and a previous study suggested that the presence and activity of calpains in muscle cells is associated with post-mortem proteolysis and meat maturation^60^. Moreover, two other CAPN family genes, *CAPN1* and *CAPN3*, were identified to affect the tenderness of breast muscle^61^.

Interestingly, we found three DMGs, *LRP8, GSK3B* and *PLXNA4*, which are related to Parkinson’s and Alzheimer’s diseases in mammals^62^,^63^,^64^,^65^,^66^. Muscle movement disorders are the main effect of age-related neurodegenerative disorders, such as Parkinson’s and Alzheimer’s diseases^67^. Our results showed that the mRNA expression level of *PLXNA4* was upregulated in the old-age group hens, and this might result in muscle weakness with an increase in age. The present study indicated that chicken might be used as an animal model of Parkinson’s and Alzheimer’s diseases in some areas.

The regulation of muscle development and composition is a complex biological process involving muscle, fat, and connective tissue. Therefore, examining regulatory networks is the preferred method of analysis^38^. In the present study, the DMGs were enriched in several predicted pathways, including the calcium signalling pathway, vascular smooth muscle contraction, the ErbB signalling pathway, focal adhesion, the Wnt signalling pathway, ubiquitin mediated proteolysis, ECM-receptor interaction, the Jak-STAT signalling pathway, the Hedgehog signalling pathway, ABC transporters, and DNA replication, which are related to muscle development, protein catabolism, energy metabolism and lipid metabolism processes. Among them, several well-known pathways related to myogenic fibre-type development and differentiation were found, including vascular smooth muscle contraction and the Hedgehog and calcium signalling pathways, and the three pathways were also found in previous studies on muscle development in chicken^26^,^38^. A previous study demonstrated that pathways related to cell junctions (ECM-receptor interaction, focal adhesion) might form a network with pathways related to lipid metabolism to influence the deposition of IMF^9^. The Wnt signalling pathway, which plays a major role in regulating carcass characteristics, is important for production traits in chickens^68^. The ErbB signalling pathway probably plays a role upstream of MAPK signalling, which is a well-known pathway affecting lipid metabolism, and is associated with skeletal muscle growth and development in chickens^26^. A previous study demonstrated that the Jak-STAT signalling pathway participates in fatty acid decomposition and, thus, influences lipid metabolism^69^. Ubiquitin-mediated proteolysis may play a crucial role in protein degradation during the ageing process, and protein degradation affects the muscle shear and, thus, changes the meat tenderness. Furthermore, DNA methylation plays an important role in the process of DNA repair after DNA damage during the ageing process in animals^70^, and this is consisted with the our present results. Additional studies of the translational and posttranslational effects will be required to complement these mRNA expression analyses. To complete the understanding of meat quality in chickens, further examination of the expression and function of the proteins encoded by the genes identified here at different age stages should be included.

In summary, the present study provides comprehensive DNA methylation profiles as well as an integrated analysis of DNA methylation and the transcriptome in the breast muscle of hens, and it also revealed potential genes and pathways related to muscle development and meat quality regulated by DNA methylation. In addition, these genes may serve as epigenetic markers for evaluating meat quality in chicken. The results of this study will contribute to our understanding of the genome epigenetic mechanism in muscle development and meat quality in different age stages and provide references for improving the quality of poultry carcasses.

## Materials and Methods

### Animal materials

All experimental protocols for the collection of animal samples were conducted in accordance with the Guidelines for Experimental Animals. And the animal experiments were approved by College of Animal Science and Veterinary Medicine, Henan Agricultural University (Zhengzhou, China). A total of twelve Gushi hens were used in this study from two age groups: 20 weeks old and 55 weeks old, representing juvenile and later laying-period hens, respectively. Each age group included six individuals, which were regarded as biological replicates. The animals were fed the same diet *ad libitum* during the experimental period, as well as in the same environment. After bleeding, 10 mL blood was collected, heparin anticoagulation, 3000 rpm centrifugation, extraction of supernatant, stored at −80 °C. The left of breast muscle tissues of the 6 individuals were flash frozen in liquid nitrogen and then stored at −80 °C until DNA and RNA extraction. The entire right breast was collected and stored at −20 °C for trait measurements.

### Measurements of the meat quality traits and serum lipid levels

Samples of the right pectoralis major muscle were homogenized using the method described by Folch^71^. The measurements and data were obtained as follows: The IMF content of the breast muscle was measured by Soxhlet extraction with anhydrous diethyl ether as described by Cui *et al*.^9^. The IFW was measured with a vernier caliper. The SF was determined for the breast muscles following Li’s method using a universal Warner-Bratzler testing machine MTS Synergie 200 (G-R Manufacturing Company, Manhattan, KS)^72^. The shear values are reported as kg of shear per cm^2^ of sample. Drip losses were estimated as described by Li *et al*.^72^. The loss was calculated as the percentage weight reduction after 48 h. The content of the TG, HDL-C and LDL-C in the solvent phase, after centrifugation, was analysed with a Triglyceride (TG enzymatic) test kit (Nanjing Jancheng Bioengineering Institute, Nanjing, China). The OD values of the samples were determined using a VersaMax microplate reader (MD, Sunnyvale, USA) according to the operation manual.

### DNA extraction and preparation

We randomly selected three 20-week-old and three 55-week-old Gushi hens as biological replicates. DNA was isolated by phenol-chloroform extraction and the integrity and purity of the DNA were evaluated by 1% agarose gel electrophoresis and spectrophotometer. The RNA samples for RNA-seq was extracted from the same individuals. The DNA from three birds within each group was mixed in equal amounts to generate a pooled sample. The DNA concentrations were measured using a Qubit DNA Assay Kit with a Qubit 2.0 Fluorometer (Life Technologies, Carlsbad, CA, USA).

### Library construction and sequencing

A total of 4.8 μg of genomic DNA spiked with 24 ng lambda DNA was fragmented by sonication to 200–300 bp with the Covaris S220 Focused-ultra sonicator (Covaris, Woburn, MA, USA) followed by end repair and adenylation. All of the DNA fragments were ligated to a sequencing adapter in which all of the cytosines were methylated. The DNA fragments were then treated with bisulfite using an EZ DNA Methylation Gold Kit (Zymo Research, Irvine, CA, USA). After treatment, the un-methylated cytosine changed into uracil (PCR amplification to T), and the methylated cytosine remained unchanged. Finally, PCR amplification was used to obtain the final DNA library.

After the library was constructed, a Qubit2.0 Fluorometer was used for the preliminary quantification. The inserted fragment length of the library was then detected using an Agilent 2100 (Agilent Technologies, Santa Clara, CA, USA), and the Q-PCR method was used to determine the effective concentration of the library. Qualified libraries were subjected to high-throughput sequencing using an Illumina Genome Analyzer II to generate 150-bp paired-end reads for the methylation profile analysis by Novogene (Beijing, China).

### Data Analysis

The raw reads produced by the Illumina HiSeq in FastQ format were first preprocessed through in-house Perl scripts. We filtered out reads that contained adapters, N (unknown bases) and those in which over 50% of the sequence exhibited a low quality value (PHRED score ≤5). At the same time, the Q20, Q30, and GC content of the data were calculated. The remaining reads that passed the filters were called clean reads, and all of the subsequent analyses were based on these.

The methylation data were aligned to the reference genome Gallus gallus-4.0 by Bismark software (version 0.12.5)^73^. The results of the sequencing and the reference genome were transformational (C to T and G to A). The transformation of the sequencing results and the genome were a pairwise comparison, which was then indexed using bowtie2^74^. The best one of the four parallel alignment results was selected as the final alignment result. The proportion of the number of aligned reads in the total number of reads was regarded as the mapping rate. The same reads that aligned to the same regions of the genome were regarded as duplicated reads. The sequencing depth and coverage were summarized using the deduplicated reads.

In the second-generation sequencing, the sequencing depth of each locus was not the same, and the proportion of methylation to unmethylation was different at different sites. However, the probability of methylation at each site obeyed the Binomial distribution. The Binomial Distribution, or the Bernoulli Experiment, which repeats n times, is the probability distribution of a discrete random variable with a wide range of applications. Based on the methylation data of the Bismarck software, the methylated cytosine and unmethylated cytosine frequencies at each site were tested for Binomial distributions in order to identify whether the site was a true methylation site. To find the exact methylation site, a set of thresholds were set during the analysis process: (1) Sequencing with a depth greater than or equal to 5 and (2) a q-value less than or equal to 0.05.

For the identified methylation sites, the methylation level (ML) was calculated as follows (mC and umC represent the number of methylated C and unmethylated C, respectively):


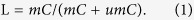


According to previous studies^75^, due to the influence of the bisulfite conversion rate, the level of methylation needed to be corrected. ML_corrected represents the corrected methylation level, r represents the bisulfite non-conversion rate, and the corrected ML was estimated as:


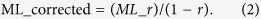


### Identification of DMRs

To determine the differential methylation regions between the two age groups, the present study used swDMR software (http://122.228.158.106/swDMR/), and a sliding-window approach (Select every 1000 bp as a window, with 100 bp as the step length) was performed to identify the differential methylation regions in the genome. Those regions, with corrected P-values less than 0.05, a read coverage greater than 5, fold-change values greater than 2 and a false discovery rate (FDR) less than 0.05, were regarded as differential methylation regions (DMRs). When the region where a DMR and a specific gene function element overlapped, the corresponding gene was selected as the DMR-related gene, namely, a differentially methylated gene (DMG).

### Functional enrichment analysis

Gene ontology (GO) enrichment and KEGG pathway analyses were conducted for the differentially methylated and expressed genes to investigate their biological processes and functions. The gene ontology enrichment analysis for the DMGs was performed using the GOseq R package^76^. KOBAS software^77^ was used to test the statistical enrichment of DMGs in the KEGG pathways. The GO terms and KEGG were corrected using the Benjamini-Hochberg method, and corrected P-values less than 0.05 were considered significantly enriched by the DMGs. The DMGs involved in the KEGG pathways related to development and metabolism were submitted to STRINGv10.0 for protein-protein interaction (PPI) network analysis (http://string-db.org/)^78^. Visualization of the PPI network was performed using Cytoscape version 3.4.0^79^.

### Bisulfite sequencing PCR

For the WGBS validation, 250 ng of the pooled genomic DNA (diluted to 250 ng/μL) from each group was treated with bisulfite sodium using the MethylEdge^®^ Bisulfite Conversion System (Promega Research, Wisconsin, USA) according to the manufacturer’s instructions. The bisulfite-treated DNA was used for PCR. Two hypermethylated regions and one hypomethylated region were selected randomly. Online MethPrimer software^80^ (http://www.urogene.org/methprimer/) was used to design the bisulfite sequencing PCR (BSP) primers (Supplementary Table S7). The BSP reaction was performed in 50 μL containing 100 ng genomic DNA, 1 μL of each primer (20 μM), 0.25 μL of TaKaRa EpiTaq HS (5 U/μL), 5 μL of 10× EpiTaq PCR Buffer (TaKaRa, Dalian, China), 5 μL of MgCl_2_ (25 mM), and 6 μL of a dNTP mixture. The PCR was performed using the following program: 94 °C for 5 min; 40 cycles of 94 °C for 1 min; 60 °C for 1 min; 72 °C for 30 s; and ending with incubation at 72 °C for 10 min. The PCR products were detected by 2% agarose gel electrophoresis and cloned into the pMD18-T vector (TaKaRa). Ten clones were selected for each gene and were subsequently sequenced by BGI (Wuhan, Hubei, China). All of the sequences were analysed by the online software QUMA (http://quma.cdb.riken.jp/)^81^. All the BSP experiments were repeated at least three times.

### Quantitative Real-time PCR (Q-PCR)

Quantitative Real-time PCR (Q-PCR) was used to measure the mRNA expression levels of the *ABCA1, COL6A1*, and *GSTT1L* genes. Total RNA was extracted from the breast muscle tissues using the TRIzol reagent (Invitrogen) and was further purified with RNeasy columns (Qiagen) according to the manufacturer’s protocol. The primers for the Q-PCR were designed using online software Primer3plus^82^ (http://primer3.sourceforge.net/webif.php) and were synthesized by Sangon biotech Co., Ltd. (Shanghai, China) (Supplementary Table S5). Each of the RNA samples was diluted to 1000 ng/uL. The cDNA was synthesized using the primeScript^TM^ RT reagent kit (Takara). Q-PCR was performed using SYBR^®^ Premix Ex TaqII (Takara) on the LightCycler^®^ 96 Real-Time PCR system (Roche Applied Science) in a 10-uL reaction volume containing 1 μL cDNA, 5 μL 2× SYBR^®^Premix Ex Taq™ II (TliRNaseH Plus) (TaKaRa), 0.5 μL each of forward and reverse primers (10 μM), and 3 μL RNase-Free Water. All of the data were normalized to the expression level of actin. The Q-PCR amplification procedure was as follows: 95 °C for 3 min; 35 cycles of 95 °C for 30 s; 60 °C for 30 s; 72 °C for 20 s; and an extension for 10 min at 72 °C. All of the Q-PCR reactions were performed in triplicate. The 2^−ΔΔCt^ method was used to determine the relative mRNA abundance^83^.

## Additional Information

**Accession codes:** The WGBS data from this study have been deposited in NCBI Sequence Read Archive with accession number SRS1788540 and SRS1793712.

**How to cite this article:** Zhang, M. *et al*. Genome-wide DNA methylation profiles reveal novel candidate genes associated with meat quality at different age stages in hens. *Sci. Rep.* **7**, 45564; doi: 10.1038/srep45564 (2017).

**Publisher's note:** Springer Nature remains neutral with regard to jurisdictional claims in published maps and institutional affiliations.

## Supplementary Material

Supplementary Information

Supplementary Dataset 1

## Figures and Tables

**Table 1:** Composition of data generated by genome-wide bisulfite sequencing.

Samples	Raw reads	Raw bases (G)	Clean reads	Clean bases (G)	Total mapped reads	Mapping rate (%)	Duplication rate (%)
55 W	282342846	35.29	279988624	35.00	139994312	78.28	13.45
20 W	275428446	34.43	271942082	33.99	135971041	75.09	17.28

**Table 2:** Genome-wide methylation levels of the two groups.

Samples	mC percent (%)	mCpG percent (%)	mCHG percent (%)	mCHH percent (%)
55 W	3.26	63.09	0.55	0.56
20 W	3.25	63.28	0.30	0.32

**Table 3:** Eighteen DMGs that overlapped with DEGs.

Gene ID	Gene Name	DMRs	Methylation Stat (55 W vs 20 W)	UP/DOWN Regulate (55 W vs 20 W)
ENSGALG00000015433	ABCA1	exon, utr5, promoter	Hyper	Down
ENSGALG00000015960	ADAMTS2	intron	Hyper	Down
ENSGALG00000000621	ANKRD47	promoter	Hyper	Down
ENSGALG00000005974	COL6A1	promoter	Hyper	Down
ENSGALG00000003439	CRY2	exon, intron	Hyper	Down
ENSGALG00000006000	EPHB2	intron	Hypo	Down
ENSGALG00000005204	GSTT1L	promoter	Hyper	Down
ENSGALG00000011762	MALT1	promoter	Hyper	Down
ENSGALG00000017130	PLEKHM2	intron	Hyper	Up
ENSGALG00000001419	PLXNA4	intron	Hyper	Up
ENSGALG00000002699	RHOG	intron	Hyper	Down
ENSGALG00000008436	RPIA	promoter	Hyper	Up
ENSGALG00000004741	SLC44 A1	intron	Hyper	Up
ENSGALG00000014525	SLC47A1	intron	Hyper	Up
ENSGALG00000015439	UBA6	exon, utr5, promoter	Hyper	Up
ENSGALG00000004583	UBE4A	intron	Hypo/hyper	Up
ENSGALG00000005475	USP5	exon, intron	Hyper	Up
ENSGALG00000002872	VAV2	intron	Hypo	Down
