# Supplementary material for: Genome-wide DNA methylation profiles reveal novel candidate genes associated with meat quality at different age stages in hens
Source: Sci Rep. 2017 Apr 5;7:45564. doi: 10.1038/srep45564 (PMC5381223; doi:10.1038/srep45564)
Supplement: Supplementary Information [file srep45564-s1.pdf]

Supplementary Information

Genome-wide DNA methylation profiles reveal novel candidate genes associated with meat quality at different age stages in hens

Meng Zhang[1], Fang Li[1], Ke-Ren Jiang[1], Dong-Hua Li[1], Rui-Li Han[1,2], Zhuan-Jan Li[1,2], Rui-Rui Jiang[1,2], Xiao-Jun Liu[1,2], Xiang-Tao Kang[1,2]&Gui-Rong Sun[1,2]

Correspondence: grsun2000@126.com.

[1]College of Animal Science and Veterinary Medicine, Henan Agricultural University, Zhengzhou, 450002, China.

[2]Henan Innovative Engineering Research Center of Poultry Germplasm Resource, Zhengzhou 450002, China.

Supplementary Data

Supplementary figures:

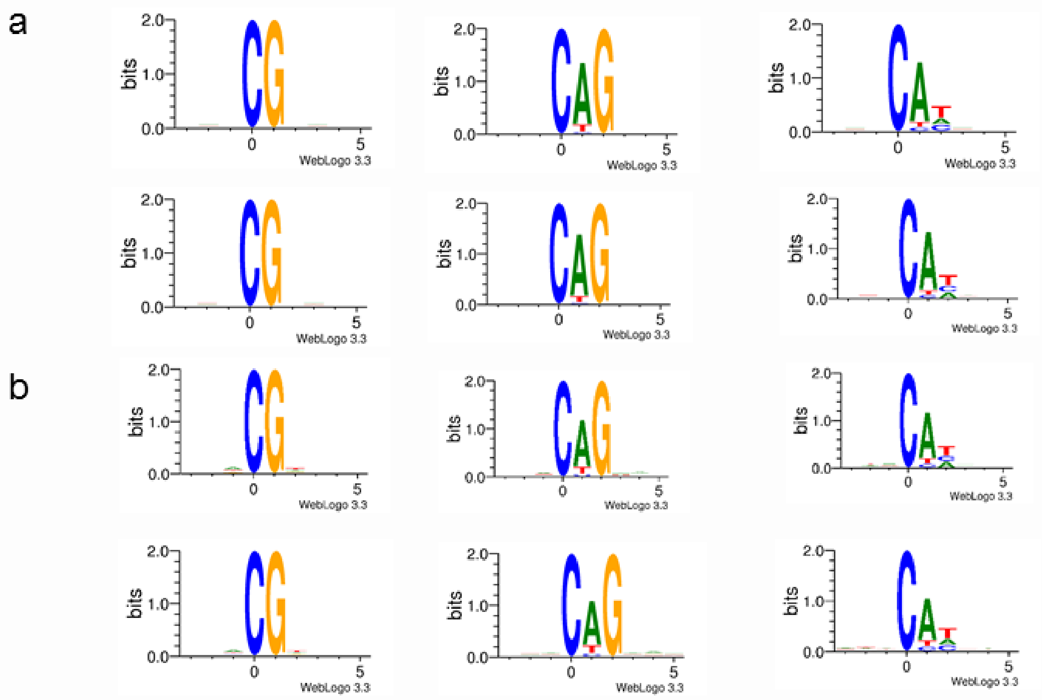

Supplementary Figure S1. Sequence preferences for methylation in various sequence contexts. 9bp base information around the positon of mCG, mCHG, mCHH at the highest or lowest methylation levels, in which the methylated cytosine is in the fourth position. (a) Regions of high methylation. (b) Regions of low methylation.

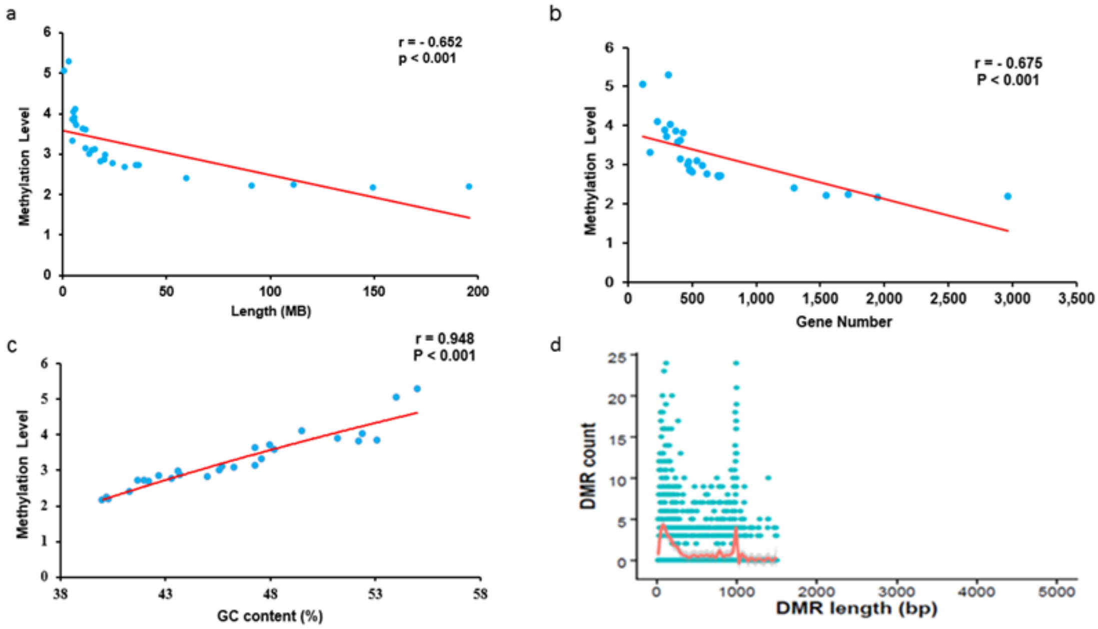

Supplementary Figure S2. Pearson's correlation between DNA methylation levels and chromosomal features; the distribution of DMRs count in length.

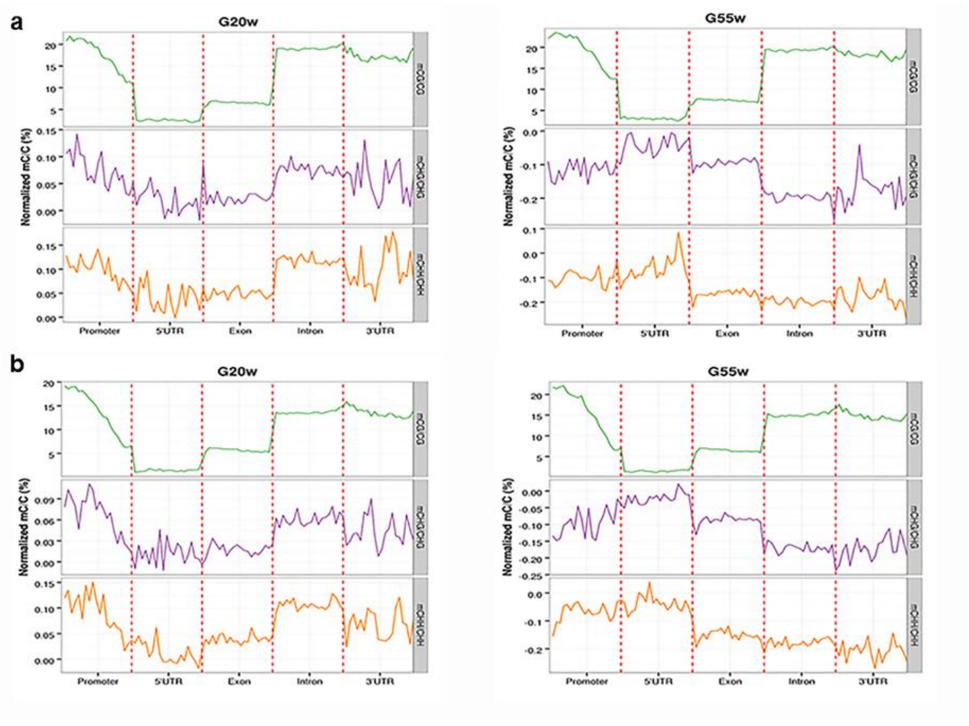

Supplementary Figure S3. Association analysis of DMGs and DEGs. DEGs

were divided into high and low-level expression types, according to their gene expression level. For each type of gene, methylation levels were analyzed in five functional element regions. (a) High-level expression of genes. (b) Low-level expression level of genes. DEG, differentially expressed gene; DMG, differentially methylated gene.

Hyper:

MYO18B

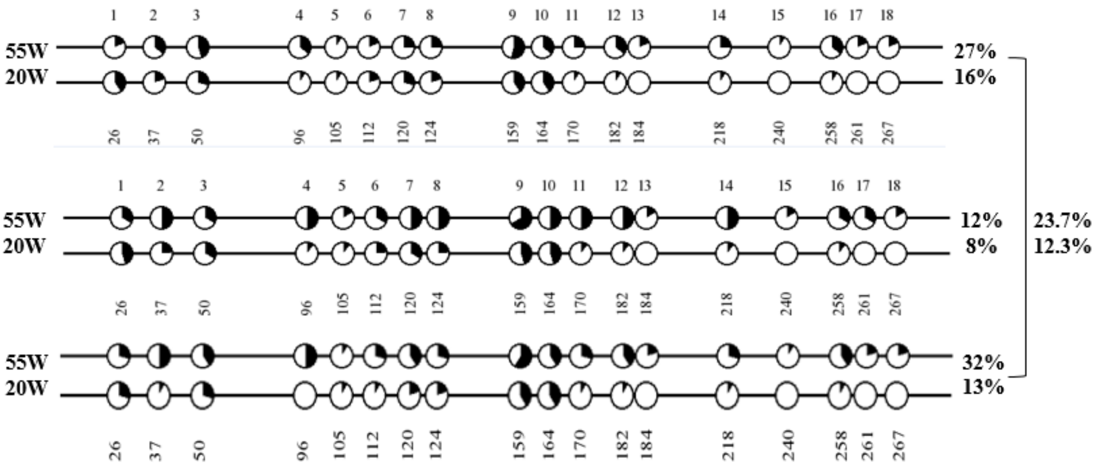

P2RY

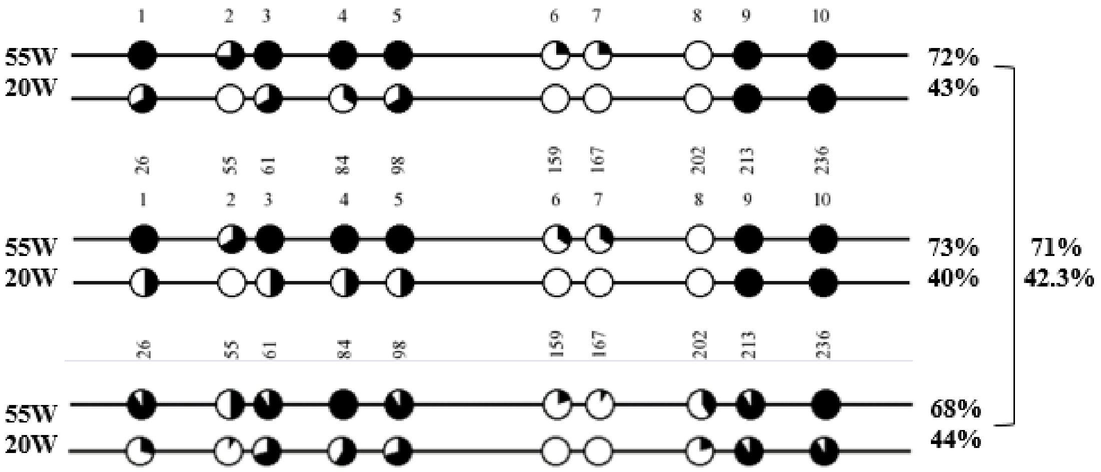

Hypo:

EPHB2

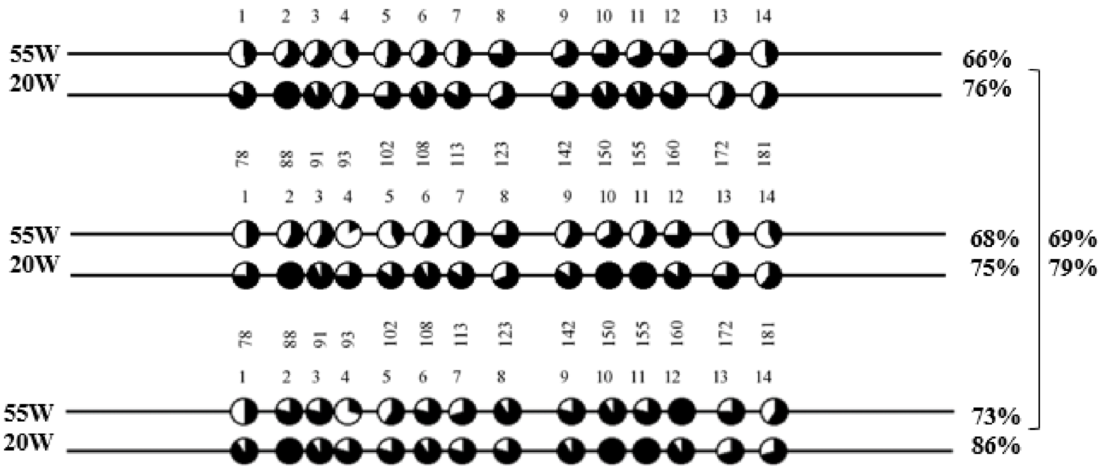

Supplementary Figure S4. The validation of CpG methylation by BSP. Two hyper (*MYO18B*, *P2RY*) and one hypor-methylated (*EPHB2*) DMRs were randomly selected. Ten subclones were selected for the BSP analysis. Solid circles represent methylated CpG sites, and open circles represent unmethylated CpG sites.

Supplementary tables：

Supplementary Table S1. Diameter and density of the breast muscle fibers of 20 and 55 weeks GuShi hens.

Items	20 Weeks	55 Weeks	P value
Diameter(μm)	30.93	33.84	0.569
Density(N/mm^2)	1140.59	905.24	0.355

Student's paired t-test (n = 6). **P < 0.01, *P < 0.05, NS: no significant difference (P > 0.05).

Supplementary Table S2. Amino acid compositions of the chest muscle of GuShi hens at 20 and 55 weeks (mg/g fresh sample).

Items	20 Weeks	55 Weeks	P value
Lysine(Lys)	21.71	21.51	NS
Phenylalanine(Phe)	8.58	8.22	NS
Methionine(Met)	6.86	6.71	NS

Threonine(Thr)	11.85	11.73	NS
Isoleucine(Ile)	9.60	9.59	NS
Leucine(Leu)	20.70	19.81	*
Valine(Val)	11.44	11.31	NS
Aspartic acid(Asp)	22.66	22.34	NS
Glutamic acid(Glu)	35.87	35.78	NS
Serine(Ser)	10.26	9.83	NS
Arginine(Arg)	15.11	15.06	NS
Glycine(Gly)	13.67	13.62	NS
Proline(Pro)	9.17	9.06	NS
Alanine(Ala)	14.12	13.91	NS
Cystine(Leu)	2.74	2.61	NS
Histidine(His)	4.34	4.41	NS
Tyrosine(Tyr)	8.37	8.27	NS
Total amino acids(TAA)	227.03	223.77	NS
Essential amino acid(EAA)	90.74	88.88	NS
Nonessential amino acid(NEAA)	136.31	134.89	NS
EAA/TAA(%)	39.97	39.72	NS
EAA/NEAA(%)	66.57	65.89	NS

Student's paired t-test (n = 6). **P < 0.01, *P < 0.05, NS: no significant difference (*P* > 0.05).

Supplementary Table S3. Fatty acid compositions of the breast muscle of GuShi hens at 20 and 55 weeks(g/100g fresh sample).

**Items**	**20 Weeks**	**55 Weeks**	**P value**
C14:0	0.80	0.93	NS
C16:0	26.67	25.00	NS
C17:0	0.20	0.20	NS
C18:0	7.70	6.73	NS
C22:0	0.20	0.10	NS
C24:0	0.17	Not detected	-
C16:1	5.03	4.60	NS
C17:1	0.27	0.20	NS
C18:1	37.63	42.27	*
C18:2	20.57	18.93	NS
C18:3	0.70	0.50	NS
C12:1	0.50	0.40	NS
SFA(%)	35.58	33.01	NS
UFA(%)	64.42	67.00	NS

SFA and PUFA are saturated and polyunsaturated fatty acids, respectively.

Student's paired t-test (n = 6). **P < 0.01, *P < 0.05, NS: no significant difference (*P* > 0.05).

Supplementary Table S4. Differentially methylated regions shared by 55W and 20W groups.

Supplementary Table S5. Primer sequences for BS-PCR and QPCR.

**Genes**	**Primer sequences (5’-3’)**	**CpGs in product**	**Product size (bp)**	T_{ann}[a] **(ºC)**
MYO18B-F[b]	ATATTGGGATTGTTTGTGTGTTAGT	18	293	58
MYO18B-R[c]	AATTTAACCACATTAAAAAAATCATC			
P2RY8-F	AAATAAATTTTGTGTTTGGGAGTAG	10	271	60
P2RY8-R	TACTCAACAAAAAAACTAAAAAAAA			
EPHB2-F	TTAGATAAAGAATAGGGGGATGGAGAA	14	273	60
EPHB2-R	CCTTTAAACTTACAACCCCAACACA			
ABCA1-F	CTTCGGGATTTCCTGATTGA		205	60
ABCA1-R	CCACTGAGCGATTTTGGATT			
COL6A1-F	GAAAGGCACCCACACTGACT		200	60
COL6A1-R	TGGCGACAGAGAACACTTTG			
GSTT1L-F	GTGCTACCGAGGAGCTGAAC		232	60
GSTT1L-R	TCGTGTGCTTCTTGGAACAG			
ACTB-F	GAGAGAAGATGACACAGATC		115	60
ACTB-R	GTCCATCACAATACCAGTGG			

[a] Annealing temperature. [b] Forward primer. [c] Reverse primer.
